# Experiences of family caregivers of older adults with stroke regarding caregiving competency: a qualitative study

**DOI:** 10.1080/17482631.2026.2759356

**Published:** 2026-11-04

**Authors:** Faezeh Norouzinezhad, Afsaneh Sadooghiasl, Eesa Mohammadi, Anoshirvan Kazemnejad

**Affiliations:** a Department of Nursing, Faculty of Medical Sciences, Tarbiat Modares University, Tehran, Iran; b Department of Biostatistics, Faculty of Medical Sciences, Tarbiat Modares University, Tehran, Iran

**Keywords:** Stroke, caregivers, competency-based education, qualitative research, family caregivers

## Abstract

**Background:**

With increasing life expectancy and the growing older adult population, stroke has become increasingly prevalent as one of the leading causes of long-term disability. A substantial proportion of the caregiving burden is borne by family caregivers. Caregiving competency plays a pivotal role in the quality of care, prevention of secondary complications, and improvement of patient outcomes.

**Purpose:**

To elucidate the experiences of family caregivers of older adults with stroke regarding caregiving competency from the caregivers’ perspective.

**Method:**

This qualitative study was conducted in Iran from 2024 to 2025 using a conventional content analysis approach based on the method proposed by Elo and Kyngäs (2008). Participants consisted of 14 family caregivers of older adults with stroke who were selected purposively. Data were collected through in-depth semi-structured interviews, and data analysis was performed in three phases: preparation, organisation, and reporting.

**Results:**

A total of 14 subcategories and 5 main categories emerged, including: Management of Safety and Motor Functioning; Control of Essential Intake and Elimination Processes; Maintenance and Promotion of Psychological and Emotional Well-Being; Preparedness and Knowledge Updating; and Support for the Recovery of Cognitive Functioning.

**Conclusions:**

Caregiving competency among family caregivers of older adults with stroke is multidimensional and extends beyond basic physical care to encompass emotional regulation, situational judgement, continuous learning, and cognitive support. In home-based care, caregivers function as active agents and continuous learners who play a complementary role in the rehabilitation process.

## 1. Introduction

Stroke is recognised as one of the leading causes of long-term disability and functional dependence worldwide (Cheng & Chau, 2017) and is increasingly acknowledged as a major challenge for healthcare systems, families, and societies (Şensöz et al., 2018). Most cases of stroke, particularly ischaemic stroke, occur among older adults (Kazemi et al., 2021). Although advances in medical care and improvements in acute stroke management have contributed to increased survival following stroke (Pucciarelli et al., 2014), a concomitant consequence has been the growing number of individuals living with varying degrees of physical, cognitive, emotional, and communicative impairments (Lutz et al., 2011). Consequently, a substantial proportion of the care required by these patients after hospital discharge is transferred to the home setting, and the primary responsibility for care falls on family caregivers or informal caregivers (Lutz et al., 2011).

The sudden onset of stroke, profound changes in family roles, and the need for rapid adaptation to new circumstances transform the caregiving experience into a multidimensional and challenging process that extends beyond routine family care (Qiu et al., 2018). This necessity has shifted scholarly attention from an exclusive focus on the negative consequences of caregiving toward the examination of the capacities and capabilities that enable effective performance of the caregiving role—a capacity that is referred to in the literature as “caregiving competency” (Barenji et al., 2013).

The concept of caregiving competency extends beyond the mere performance of caregiving tasks and refers to a multidimensional set of knowledge, practical skills, situational judgement, problem-solving ability, emotional adaptability, and the capacity to manage complex caregiving situations (Barenji et al., 2013). This concept is associated not only with the quality of care provided but also with caregivers’ psychological well-being, the sustainability of the caregiving role, and patients’ functional outcomes (Panzeri et al., 2019). Despite the importance of this construct, most existing studies have primarily focused on the adverse consequences of caregiving, such as caregiver burden, stress, or psychological distress, whereas the empowering dimensions of the caregiving experience—particularly the development and meaning of competency among family caregivers of individuals with stroke—have received less attention (Farombi et al., 2024). Therefore, a comprehensive understanding of this phenomenon cannot be achieved solely through quantitative measurement; rather, it requires exploration of caregivers’ lived experiences and the meanings they attribute to their own capabilities within the caregiving role. Qualitative research provides an opportunity to uncover the process of competency development, associated challenges, adaptive strategies, and facilitating or inhibiting factors from the caregivers’ own perspectives (Creswell & Clark, 2017; Safadi et al., 2011).

Understanding the experience of competency among family caregivers of individuals with stroke can contribute to the development of targeted educational and supportive interventions, improvement of pre-discharge preparation programmes, strengthening of family-centred care, and enhancement of health outcomes for both patients and caregivers (Qiu et al., 2018). Such knowledge may also help identify gaps between caregivers’ actual needs and the services provided by the healthcare system, thereby offering a foundation for the design of evidence-based supportive policies and programmes (Bakas et al., 2022). Accordingly, the present study was conducted to elucidate and interpret the meaning of caregiving competency as experienced by family caregivers of older adults with stroke.

## 2. Methods

### 2.1. Study design

This qualitative study was conducted using a conventional qualitative content analysis approach. Conventional content analysis is appropriate when the aim is to derive concepts, definitions, and dimensions of a phenomenon directly from the data and the lived experiences of participants, without imposing predetermined theoretical categories (Hsieh & Shannon, 2005; Marvasti, 2019). To avoid remaining at the level of surface description, categories were developed through constant comparison and abstraction of codes across interviews, and the research team repeatedly returned to the data to ensure that categories reflected participants' accounts. Existing frameworks of caregiving competency were not used as coding schemes. They were used only as sensitising concepts during the interpretation of the categories, after the categories had emerged, and are discussed in relation to our findings in the Discussion (Graneheim et al., 2017).

While prior work on caregiving competency exists in other disease contexts (e.g. spinal cord injury, dementia, palliative care) (Jeyathevan et al., 2019; Jung et al., 2022; Wang et al., 2025), the contextual specificity of stroke caregiving among older adults in the Iranian cultural setting warranted an inductive approach that privileges participants' lived experiences over a priori frameworks.

### 2.2. Participants and sampling

The study participants consisted of family caregivers of older adults with stroke. Older adults were defined as individuals aged 60 years and older, consistent with the World Health Organization definition and the Iranian National Elderly Health Programme criteria (WHO, 2024; Sharifi et al., 2016).

Inclusion criteria were: having direct responsibility for caring for the patient at home; having at least one month of continuous caregiving experience; confirmation of the patient's stroke diagnosis by a physician; patient age of 60 years or older; willingness to participate in the study; being aged 18 years or older; ability to communicate effectively in Persian; and ability to clearly articulate personal experiences.

Purposive sampling with maximum variation was employed. Participants were purposefully selected to ensure diversity in caregiver age, gender, educational level, employment status, and relationship to the patient, duration of caregiving, and patient age and gender. Participants were recruited through referrals from therapists, healthcare providers, and rehabilitation centres. To achieve maximum variation, recruitment continued until the sample reflected a broad range of caregiver characteristics, including both male and female caregivers (5 male, 9 female), ages ranging from 21 to 62 years, various educational levels (primary school to master's degree), and caregiving durations from 2 months to 5 years. Table I presents the demographic characteristics of the participants. The research setting included hospitals, neurology wards, and rehabilitation centres, which served as referral sites for patients and their families.

**Table I.** Demographic characteristics of the participants.

Participant	Gender	Age (years)	Marital Status	Education	Employment status	Relationship to patient	Duration of illness	Patient age (years)	Patient gender	Interview duration (min)	Interview type
1	Female	50	Single	Middle School	Unemployed	Daughter	5 years	82	Female	40	Face-to-face, patient's home
2	Female	48	Married	Bachelor's Degree in Physical Education	Teacher	Daughter	1 year	84	Female	45	Face-to-face, patient's home
3	Female	51	Married	Tenth Grade Secondary School	Unemployed	Spouse	1 year and 6 months	61	Male	67	Hospital
4	Female	28	Single	Master's Degree	Unemployed	Daughter	2 months	62	Female	55	Hospital
5	Male	30	Married	Bachelor's Degree	Employed	Son	4 years and 4 months	61	Male	66	Hospital
6	Male	40	Married	High School Diploma	Farmer	Son	8 months	71	Male	61	Hospital
7	Female	52	Married	Bachelor's Degree	Language Teacher	Sister-in-law	8 months	62	Female	58	Rehabilitation Centre
8	Male	21	Single	High School Diploma	Self-employed	Grandson	3 months	65	Male	35	Hospital
9	Female	62	Married	High School Diploma	Homemaker	Spouse	5 years	78	Male	82	Telephone
10	Female	25	Single	High School Diploma	Unemployed	Daughter	4 months	61	Female	52	Hospital
11	Male	31	Married	Associate Degree	Self-employed (Electrical Services)	Son	6 months	65	Male	48	Hospital
12	Male	59	Married	Primary School	Self-employed	Son	6 months	86	Male	77	Hospital
13	Female	37	Single	Master's Degree	University Lecturer	Daughter	1 year and 2 months	63	Female	50	Hospital
14	Female	35	Married	High School Diploma	Homemaker	Daughter	4 months	73	Female	40	Telephone

### 2.3. Data collection

Data were collected through in-depth, individual semi-structured interviews. Following the acquisition of both written and verbal informed consent, interviews were initiated and continued until data saturation was achieved. A total of 14 interviews were conducted. Data saturation was determined when no new codes or categories emerged from consecutive interviews. In practice, after the 11th interview, no new substantive codes were identified, and subsequent interviews (12th–14th) served to confirm the stability and completeness of the emergent categories. The research team met after each interview to review coding progress and discuss whether saturation had been achieved.

At the beginning of each interview, general questions were asked to enable the researcher to become more familiar with the participants and to establish a friendly and trusting atmosphere. Subsequently, more specific questions aligned with the study objectives were introduced. The duration of interviews ranged from 35 to 82 minutes (mean = 55 minutes). All interviews were conducted and audio-recorded in Persian by the first author, a doctoral student in nursing with experience in qualitative interviewing.

Although all participants had been providing care at home for at least one month, some interviews were conducted in hospital or rehabilitation centre settings when caregivers accompanied patients for follow-up appointments, rehabilitation sessions, or acute care visits. Conducting interviews in these settings ensured privacy and minimised disruption to caregiving responsibilities.

Clarifying, probing, and in-depth follow-up questions were used to guide and further explore participants’ responses. During the interviews, the researcher paid close attention to participants’ emotions, facial expressions, tone of voice, and other non-verbal cues. At the end of each interview, the participant was thanked and asked whether there was anything else they would like to share about their experiences of caring for a person with stroke. The participant was also asked about their availability and preferences regarding a subsequent appointment for data verification or a follow-up interview. At the earliest opportunity following each interview, the recorded material was transcribed verbatim, and the original audio files were stored in confidential folders.

The semi-structured interview guide is provided as Supplementary File 1.

### 2.4. Data analysis

Data were analysed using the conventional content analysis approach proposed by Elo and Kyngäs (2008), consisting of three phases: preparation, organisation, and reporting (Elo & Kyngäs, 2008). During the preparation phase, interview transcripts were read repeatedly to achieve a comprehensive understanding of the data. Coding was then initiated. At this stage, each transcript was reviewed sentence by sentence, and meaning units (i.e. any sentence relevant to the research question) were identified and underlined. An appropriate open code was assigned and written alongside each meaning unit.

During the organisation phase, Coding was conducted by the first author (F.N.) and independently reviewed and confirmed by the second author (A.S.) and third author (E.M.), all of whom had experience in qualitative research. Any disagreements regarding coding were resolved through discussion and consensus and, when necessary, through consultation with external reviewers and experts in qualitative content analysis. After several interviews had been analysed, transcripts were reread and similarities and differences among the open codes were compared to determine which codes could be grouped together. Codes from each interview were compared both within and across previous and subsequent interviews to identify commonalities and distinctions. Similar codes were grouped into subcategories through iterative consensus meetings. Subcategories were subsequently abstracted into main categories based on their conceptual relationships and shared meanings, with the research team engaging in ongoing dialogue to ensure that the abstraction process remained grounded in the data.

In the third phase, reporting, the data analysis procedures and methodological processes, including coding, category extraction, and the development and interpretation of the main categories, were presented in the Findings section. Table II illustrates the process through which the category Management of Safety and Motor Functioning was developed.

**Table II.** Development process of the category “Management of Safety and Motor Functioning”.

Meaning Units	Codes	Subcategories	Main category
“We had to pay close attention to him and to the surrounding environment. We had to make sure that nothing happened to him again. We had to prevent him from falling.” (P3)	Prevention of patient falls	Environmental safety and injury prevention	Management of Safety and Motor Functioning
“We repositioned him every two hours. We placed a pillow under his leg because it was numb. We also put a pillow under his arm. We kept repositioning him. We were very afraid of pressure ulcers.” (P6)	Repositioning the patient to prevent pressure ulcers		
“I tried to encourage her to walk and help her do so.” (P10)	Educating and encouraging the patient to walk	Maintaining joint health and range of motion	
“I put the cane in her hand because my mother walks with a cane. I hold her other hand and support her under the arm while helping her walk.” (P13)	Assisting the patient with walking at home		
“Then we used to massage him. Whenever we massaged him, it seemed to make him feel relaxed.” (P5)	Massaging the limbs		
“We helped him perform arm and leg exercises. Overall, we were very active in supporting her exercises because we wanted him to regain mobility as quickly as possible.” (P11)	Assisting with movement of the patient’s limbs		

### 2.5. Positionality statement

The research team consisted of nursing academics with backgrounds in gerontological nursing, qualitative research, and biostatistics. The first author (F.N.) is a doctoral candidate in nursing with training in qualitative interviewing. The second author (A.S.) is an associate professor of nursing specialising in geriatric care and qualitative methods. The third author (E.M.) is a professor of nursing with extensive expertise in qualitative content analysis. The fourth author (A.K.) is a professor of biostatistics. To minimise the influence of researchers’ preconceptions on the analytical process, an inductive approach was employed, and efforts were made to bracket researchers’ initial assumptions as much as possible during the analysis so that the findings remained grounded in participants’ narratives and experiences. In addition, member checking and external checking were used to enhance the credibility of the findings, and an audit trail was maintained throughout all stages of the study.

### 2.6. Translation and language consideration

All interviews were conducted in Persian. The interviews were transcribed verbatim in Persian by the first author. Member checking was conducted with seven participants in Persian during the study. Following completion of the study, the manuscript was initially written in Persian. For publication purposes, selected quotations and the coding framework were translated into English by the first author and reviewed by a native English-speaking translator who was proficient in both Persian and English. The initial and final versions of the manuscript in both Persian and English were reviewed by all authors, who confirmed the accuracy and fidelity of the translation.

### 2.7. Ethical consideration

This study was approved by the Research Ethics Committee of Tarbiat Modares University under the ethics code IR.MODARES.REC.1401.252. After receiving a full explanation regarding the nature and objectives of the study, participants completed a written informed consent form. Voluntary participation and the right to withdraw from the study at any time were ensured. Confidentiality of the data, anonymity in the dissemination of findings, and permission for audio recording of interviews were also maintained throughout the study. This study was conducted in accordance with the Declaration of Helsinki.

### 2.8. Trustworthiness of the data

The trustworthiness of the data was ensured according to the four criteria proposed by Guba and Lincoln (1994), namely credibility, dependability, confirmability, and transferability (Guba & Lincoln, 1994). To enhance credibility, several strategies were employed, including prolonged engagement with the data (for more than eight months), peer debriefing and review by research team members and colleagues, as well as maximum variation sampling in the selection of participants (with respect to age, gender, duration of the older adult’s illness, employment status, educational level, and marital status of caregivers). To ensure a shared understanding of the codes derived from the interviews, member checking was conducted with seven participants. Data analysis and coding procedures were supervised and confirmed by members of the research team (the second and third authors), who had substantial experience in conducting qualitative research. To enhance transferability, all stages of the study, particularly data collection, data analysis, and the development of the main categories, were documented comprehensively and in detail to facilitate external auditing by readers and other researchers. External reviewers also monitored the accuracy and rigour of the research process.

## 3. Results

A total of 14 family caregivers of older adults with stroke participated in this study. The caregivers included 9 women and 5 men, aged 21 to 62 years (mean = 40.4 years). Their relationships to the patients included daughters (*n* = 6), sons (*n* = 4), spouses (*n* = 2), sister-in-law (*n* = 1), and grandson (*n* = 1). The duration of caregiving ranged from 2 months to 5 years. Table III summarises the key characteristics of the patients, including stroke type, time since stroke, level of functional impairment, and cognitive or communication difficulties.

**Table III.** Key characteristics of patients.

Participant	Stroke type	Time since stroke	Functional impairment (mRS)	Cognitive impairment	Communication impairment
1	Haemorrhagic	5 years	3 (moderate)	Yes (memory)	No
2	Ischaemic	1 year	3 (moderate)	Yes (memory)	No
3	Haemorrhagic	1 year 6 months	4 (moderately severe)	Yes (memory)	Yes (aphasia)
4	Haemorrhagic	2 months	5 (severe)	Yes (memory)	Yes (aphasia)
5	Ischaemic	4 years 4 months	3 (moderate)	No	Yes (aphasia)
6	Ischaemic	8 months	4 (moderately severe)	Yes (memory)	Yes (aphasia)
7	Haemorrhagic	8 months	5 (severe)	Yes (memory	Yes (aphasia)
8	Ischaemic	3 months	3 (moderate)	No	No
9	Haemorrhagic	5 years	5 (severe)	Yes (memory)	Yes (aphasia)
10	Ischaemic	4 months	4 (moderately severe)	Yes (memory)	Yes (aphasia)
11	Haemorrhagic	6 months	4 (moderately severe)	No	No
12	Ischaemic	6 months	5 (severe)	Yes (memory)	Yes (aphasia)
13	Ischaemic	1 year 2 months	4 (moderately severe)	Yes (memory)	Yes (aphasia)
14	Ischaemic	4 months	4 (moderately severe)	Yes (memory)	Yes (aphasia)

The majority of patients (*n* = 8) had ischaemic stroke, while 6 patients had haemorrhagic stroke. Time since stroke ranged from 2 months to 5 years. Functional impairment, assessed using the Modified Rankin Scale (mRS), ranged from moderate to severe (mRS 3–5), indicating that all patients required substantial assistance with daily activities (Banks & Marotta, 2007). 11 of 14 patients had some degree of cognitive or communication impairment, including aphasia (*n* = 10), memory problems (*n* = 11). These characteristics substantially shaped the nature and intensity of caregiving demands and, consequently, the competencies that caregivers developed and enacted.

Analysis of the qualitative data resulted in the identification of 93 initial codes, 14 subcategories, and 5 categories (Table IV).

**Table IV.** Categories and subcategories derived from the qualitative analysis.

Main categories	Subcategories
**Management of Safety and Motor Functioning**	Environmental safety and injury prevention
	Encouragement and assistance with mobility
	Maintaining joint health and range of motion
**Control of Essential Intake and Elimination Processes**	Attention to and provision of nutritional needs
	Attention to and assistance with elimination needs
	Management and provision of medication-related needs
**Maintenance and Promotion of Psychological and Emotional Well-Being**	Efforts to maintain the patient’s calmness and happiness
	Expressing affection toward the patient
	Patient, respectful, and supportive companionship
**Preparedness and Knowledge Updating**	Vigilance and Follow-Up of Complications and Acute Problems
	Monitoring the patient’s blood pressure under different conditions
	Continuous Learning and Updating
**Support for the Recovery of Cognitive Functioning**	Efforts to establish communication
	Strengthening cognitive abilities

In presenting the findings, caregiving activities were interpreted as reflecting competency when caregivers assessed the patient's condition, adapted their actions to it, anticipated risks, or learned in response to a problem, rather than performing a prescribed task.

### 3.1. Management of safety and motor functioning

This category represents caregivers’ competency in ensuring the physical safety of older adults and supporting their motor functioning. This competency enables caregivers not only to perform routine safety measures but also to identify potential risks and devise preventive and supportive measures according to the patient’s condition and environmental circumstances. They pursue and implement these measures through three characteristics: environmental safety and injury prevention, encouragement and assistance with mobility, and maintenance of joint health and range of motion.

#### 3.1.1. Environmental safety and injury prevention

Caregivers adopt measures to ensure environmental safety and prevent injuries in the home environment in order to reduce the likelihood of falls and other physical injuries. In this regard, fall prevention is one of their main concerns, and in different situations, particularly during nighttime rest, bathing, and patient transfers, they pay close attention to maintaining the patient’s safety.

Drawing on their experience, knowledge of the patient’s motor limitations, and awareness of the consequences of immobility, caregivers employed various strategies, such as using an appropriate bed, placing pillows, using a shower chair, regularly repositioning the patient, and seeking help from other family members. This characteristic indicates that, when faced with caregiving problems, they did not merely follow fixed instructions; rather, they adapted their knowledge to the patient’s actual circumstances and made situation-based decisions to prevent injury.

For example, Participant 5 stated:

“More than anything, we had to be careful that the patient did not fall. I remember the first night, we got a hospital bed and brought it home. I put several pillows behind his head. At one point, I was getting sleepy, and I saw him falling from the side of the bed. I quickly caught him, and I sat there until morning so that he would not fall.” (P5)

In addition, the repositioning of the patient every two hours, as reported by P3, P4, and P6, indicates caregivers’ awareness of the consequences of immobility and their efforts to prevent pressure ulcers.

“We were careful that he would not develop bedsores and that he would be repositioned every two hours. We repositioned him every two hours and were very careful.” (P3)

“I tried to turn her every two hours so that she would not develop bedsores.” (P4)

“At that time, my father was completely bedridden. If we did not move this arm, it would remain in the same position until morning. He could not move at all. We had to stay awake. For exactly two months, this was what we did. We turned him to one side every two hours. We were very afraid of bedsores.” (P6)

In the case of P14, involving several family members can also be considered a form of problem-solving for simultaneously managing patient safety and the physical burden of caregiving.

“All three of us would bathe my mother together. Because you really have to be very careful that she does not fall or that nothing happens to her. On the other hand, when there are several people, the work becomes easier. The pressure is not only on one person.” (P14)

Therefore, caregivers’ approach to existing and potential problems related to possible injuries indicates the use of problem-solving strategies and the application of knowledge.

#### 3.1.2. Encouragement and assistance with mobility

In the third characteristic, caregivers did not merely rely on direct recommendations to increase the patient’s motivation to exercise; rather, by recognising the patient’s interests, emotional relationships, and personal goals, they used meaningful motivators to encourage the patient to engage in activity.

In this regard, one participant stated:

“We encouraged him to be active in any way we could. We also prepared him mentally. We motivated him. My father loves his grandchild. And I am his eldest child. He used to say, ‘I love it when my grandchild tells me, “Grandpa, let’s go have some ice cream.”’ When I mentioned this to him, especially at the beginning, it had a great effect. It was as if it motivated him to be more active and do his exercises.” (P11)

Furthermore, when faced with the risk of falls and mobility limitations, caregivers redesigned the home environment according to the patient’s abilities. Participant 4’s action of arranging chairs at short intervals throughout the home represents an example of situation-based problem-solving. In this way, while enabling the patient to walk, the caregiver limited the walking path to safe areas and created the possibility of rapid intervention if the patient became tired or lost balance. This characteristic indicates that the caregiver continuously observed the patient’s condition, anticipated risks, and adjusted the environment accordingly.

“I wanted to help her walk, so throughout the house, I placed chairs about 10 centimeters apart. I basically arranged chairs all over the house. For example, I would hold one side of her. As soon as I saw that he was tired or wanted to throw herself down, I would quickly turn her toward a chair. I never let her walk outside that area of chairs.” (P4)

In managing safety and mobility, caregivers did not apply fixed instructions; they adapted their knowledge to the patient's condition and the home setting. After nearly losing the patient from the bed at night, P5 stayed beside him until morning. P3, P4 and P6 repositioned patients every two hours because they understood the consequences of immobility. P14 involved three family members in bathing to manage both the risk of falls and the physical burden of care. In maintaining range of motion, P11 installed a ceiling pulley and a wall bar, and P7 recorded the physiotherapist's exercises on video and supplemented them with exercises found online. Caregivers also adapted their approach to the individual patient. P11 used the patient's love for his grandchild to motivate him to exercise, and P4 placed chairs at short intervals throughout the house so that the patient could walk within a safe area with a place to rest.

These accounts show caregivers anticipating risks, redesigning the environment, seeking out knowledge and creating practical solutions. This reflects problem-solving and situational judgement based on knowledge, rather than the mere performance of safety routines.

#### 3.1.3. Maintaining joint health and range of motion

The second characteristic of competency in safety management is related to joint health and range of motion. Caregivers, aware of consequences such as joint stiffness and muscle weakness, use methods such as massage, moving the limbs, and performing mobility exercises to maintain joint health. This characteristic indicates that caregivers’ knowledge does not remain merely at the level of theoretical awareness and self-efficacy but is manifested in practical and effective behaviours for maintaining and promoting health. It is noteworthy that, before acquiring this competency, they used physiotherapy training, watching educational videos, and preparing or making simple devices to facilitate exercises as forms of self-directed learning.

For example, Participant 11 stated:

“... I had bought one of those physiotherapy pulleys for him and hung it from the ceiling so that he could move his arms. He would move his legs, bend them and straighten them. Or, for example, we had installed a bar on the wall. We had actually done this recently, before coming here, so that he could hold onto the bar, stand up, and sit down.” (P11)

“We were in the ward for two days when the physiotherapist came. I recorded a video and did the exercises with her. The lady who came to our home for physiotherapy also provided 20 sessions of physiotherapy at home. There were also some exercises that I knew, and I had found some exercises on the internet as well. I worked with her according to those exercises.” (P7)

Therefore, this finding indicates that, in maintaining range of motion, caregivers were not merely implementers of therapeutic recommendations; rather, by observing the patient’s condition and using various resources, they transformed their knowledge into practical and sometimes creative strategies.

### 3.2. Control of essential intake and elimination processes

This category represents how caregivers address and plan for the control of essential intake and elimination processes, including nutrition, elimination, and medication. These controls do not merely involve implementing predetermined instructions; rather, caregivers assess needs, identify problems, and make decisions based on their experiences or previous learning. Thus, caregivers gradually acquire knowledge and skills through experience and training from healthcare professionals, continuously assess the patient’s condition, and select, implement, and adjust caregiving strategies according to the patient’s abilities, limitations, and changes in condition. In this process, situational judgement, experiential learning, and acquired skills enable caregivers to identify everyday caregiving problems and develop solutions appropriate to the circumstances. Therefore, this category has a dynamic and problem-oriented nature.

#### 3.2.1. Attention to and provision of nutritional needs

In the area of nutrition, caregivers modify the type, consistency, volume, and method of food provision according to the patient’s chewing and swallowing difficulties and abilities. Participant 13 described the gradual process of adapting food to the patient’s abilities:

“During the first three months, she could not swallow at all. We gave her porridge or whatever it was through a syringe, only through a syringe. We mixed it and diluted it before giving it to her. After three months, when we took her [to the doctor], her swallowing had improved. The doctor said to make her food a little thicker so that it could help with her swallowing and so that she could chew and remember how to chew. Because the right side of her body was still paralysed, we fed her from one side. She couldn’t [eat properly], and the food would spill. So, we gradually gave her food from that side. We would chew the food with our own teeth in front of her so that she could watch us. We would tell her, ‘Mom, look at us,’ and she would watch us and imitate us. Like a child, she did not know how to swallow either, and she gradually learned.” (Participant 13)

Caregivers also determined the next stage of feeding based on the patient’s response. Therefore, caregiving was not performed as a fixed process; rather, it progressed through assessment of the patient’s response and continuous adaptation to the patient’s abilities. Participant 7 stated:

“Her doctor had told us to first give her water with a spoon. If we gave her water with a spoon and she was able to swallow it... We would just give her the water slowly so that it would not go into her throat too quickly and she could swallow it... ‘Give her a glass and let her hold it. If she can drink it... if she cannot, use a straw.’”

#### 3.2.2. Attention to and assistance with elimination needs

To control and meet the older adult’s elimination needs, caregivers undertake measures such as accompanying the patient to the toilet and assisting with hygiene, maintaining complete hygiene during diaper changes, and managing the urinary catheter. Considering the patient’s limitations and awareness of potential risks, caregivers regularly integrated preventive measures into their caregiving routines.

“Well, honestly, at that time, we emptied his urine bag every hour or so. We did not let it stay full; we emptied it frequently. We also changed the catheter itself every week so that it would not become infected.” (P14)

They emphasised that diaper changes should be performed with complete hygiene, the use of gloves, and appropriate disinfection to prevent infection and damage to the patient’s skin. In this regard, Participant 12 stated:

“These things are not trivial, and we have to be very careful. His clothes, his wipes, when you want to change him, you absolutely have to wear gloves, and there must be very thorough disinfection, because you cannot take him outside to wash him. Changing a diaper is very troublesome. Cleaning him is very important and requires a lot of special care. Gloves must be used, and disinfection must be done, because if even the smallest germ enters the tract, then, as you know, he will get an infection, and there is nothing we can do.”

Caregivers addressed elimination needs not as a fixed hygiene routine but as a risk-prevention task that they planned and adapted to the patient's condition and the home setting. They set their own preventive schedules and linked them to anticipated complications. For example, P14 emptied the urine bag frequently so that it would not remain full and changed the catheter weekly “so that it would not become infected.” Caregivers also reasoned about why specific measures mattered. P12 explained that, because the patient could not be taken elsewhere to be washed, thorough disinfection and glove use were essential to prevent infection of the urinary tract and skin damage.

These accounts show that caregivers anticipated infection risk, translated their knowledge into context-specific preventive practices, and adjusted hygiene care to the constraints of home-based care. This reflects knowledge, practical skill and situational judgement rather than the mere performance of a routine task.

#### 3.2.3. Management and provision of medication-related needs

To manage and meet medication-related needs, caregivers undertook measures such as administering medications on time and according to the physician’s instructions, being aware of the names and effects of medications, ensuring that the patient took the medications completely, becoming familiar with medications through explanations provided by nurses, and monitoring for the occurrence of drug interactions. Patients usually take several medications simultaneously, each with its own specific administration time and dosage. To reduce the likelihood of forgetfulness and errors, caregivers used resources available within the family and employed strategies such as simple reminder tools and family coordination. Participant 4 stated:

“Well, I had set alarms for all of her medications... I gave her pantoprazole in the morning... His insulin, for example, was at this time, so I would go when the alarm went off so that I would not make a mistake. I had written everything down.”

Participant 7 also explained:

“We created a family group and added everyone to it, and we put the medications and medication times in it so that everyone would be aware... We had written down her medications and put them on the refrigerator so that everyone could see them.”

These actions indicated that acquiring knowledge about medications and understanding their uses and effects was also part of the caregivers’ learning process.

One caregiver stated:

“Well, the doctor told me about her medications, and I learned them. Now I know, for example, that this is her heart medication. This furosemide is for her urination, this pill is for her cholesterol, this one is for her kidney, these are for his pain, and this one is for her sleep. The doctor said her medications would not change and that he should take them for the rest of her life. This one is to prevent blood from accumulating in her brain.” (P1)

Caregivers also followed the patient’s medication regimen by obtaining information about how and when the medications should be taken. Participant 7 stated:

“Then, for example, we looked into why she had to take this medication half an hour earlier. For example, we found out that it was a stomach medication and that she had to take it half an hour earlier so that her stomach would not be irritated. For example, we learned that Plavix was very important and had to be taken on time. So, we made sure of that...”

In some cases, caregivers monitored whether the patient took the medications completely and, if the patient did not take them, changed the method of administering the medication. Participant 2 stated:

“But after the second stroke, she would keep the pills in her mouth or take them out and throw them away. So, we had to crush the pills, put them in a glass with water, and give them to her because she had to take her medications...”

Across nutrition, elimination and medication, caregivers did not simply carry out instructions; they observed how the patient responded and adjusted their approach accordingly. In feeding, P13 moved from syringe feeding to thicker food as swallowing improved, and modelled chewing in front of the patient so that she could imitate it. P7 followed the physician's stepwise advice, testing swallowing with water given by spoon and then moving to a glass or straw according to the patient's response. In medication management, caregivers built their own safeguards against error, such as phone alarms (P4), a family group chat and a list on the refrigerator (P7). They also asked why a drug had to be taken at a particular time (P7) and changed the method of administration when the patient spat out the pills (P2).

These examples show caregivers observing, interpreting the patient's response and modifying their actions. This reflects situational judgement and problem-solving, supported by knowledge gained from health professionals and from experience.

### 3.3. Maintenance and promotion of psychological and emotional well-being

Maintenance and promotion of patients’ psychological and emotional well-being constituted another important dimension of caregivers’ competency. Caregivers made specific and thoughtful efforts to maintain the patient’s calmness and happiness through strategies such as expressing affection, providing patient and respectful companionship, and showing patience in interactions with the patient. In this regard, caregivers used strategies such as conversation and verbal support, changing the environment, maintaining contact with close relatives, music, humour, watching films, viewing family photographs, and expressing affection. The selection of these strategies varied according to the patient’s emotional state, responses, and preferences.

For example, despite the difficulties involved in transferring the patient in a wheelchair, one caregiver continued to arrange outings to improve the patient’s mood:

“We used to take her outside. Every Friday, we would go out, even though she was in a wheelchair and it was difficult. We would go by car because we wanted her mood to get better... Then we would take her to the park. We would take her there so that her mood would be excellent and she would feel good.” (P13)

Caregivers also adapted emotional interventions to patients’ preferences by recognising their interests. Participant 10 stated:

“I would buy books for her, the genres she liked herself... the songs she liked herself... It seemed to make her feel better... I would do whatever was necessary for her, whatever would make her feel good and improve her mood... It really had an effect on her.”

Maintaining social connectedness and a sense of belonging was also part of this process. By involving the patient in family gatherings and providing affection and support from family members, caregivers attempted to prevent isolation and feelings of rejection. Participant 9 stated:

“The children gather around him and help him. They show him affection... in a way, he does not feel rejected... They dress him in his best clothes and bring him into the gathering... We always try to have him among everyone... He also likes it when guests come. His sister came from Tabriz to visit him; his eyes light up... He laughs with her. He talks and is happy.”

Caregivers’ emotional adaptability to the patient’s condition was particularly evident in how they adjusted the timing and type of interventions. By identifying individuals or situations that could cause stress, they took preventive measures to avoid upsetting the patient and to modify the caregiving environment accordingly. Participant 10 stated:

“People whom she did not like, not at all... We would even tell them not to come... We did not let them [come]. Even when they called for any reason, we would not answer.”

In relation to patient and respectful companionship and maintaining patience when dealing with the patient’s condition, Participant 5 described caregiving as challenging and sometimes exhausting, noting that it could occasionally cause him to become frustrated.

He emphasised that patience and tolerance were key to successfully navigating this process. he recommended that others use humour and flexibility to facilitate communication with the patient and respond to the patient patiently, even when the patient was irritable. he stated:

“One time I got really upset and told him, ‘Am I doing this for myself? I’m doing it for you. You want to do it, do it; you don’t want to, don’t do it.’ Then I left the house. I went to the end of the street and sat there for ten minutes, then I came back. I went back to his exercises again.” (laughing)

Similarly, some participants accompanied patients during prayer times and religious practices. For example, Participant 13 explained that during Ramadan and religious occasions, they tried to accompany the patient in religious activities. She also described how, by adapting to the circumstances and developing creative solutions, such as placing food beside the patient or giving her the Qur’an to touch and look at, she was able to involve the patient in her spiritual experience, even though she was not fully able to perform religious practices. She stated:

“But, for example, it was Ramadan at that time, and my mother had a special attachment to it. Every year during Ramadan, she would go to the mosque after iftar, even at suhoor and on the nights of Qadr. She would go to the mosque, and it reminded her of herself. She would see us breaking our fast and praying, and she would cry. We would come to her, and of course she had to eat her food. At first, she would not eat and would say, ‘I want to fast too.’ I would tell her, ‘Mom, you are fasting now.’ We would prepare food for her, too, and when it was time for iftar, we would bring it to her as well. I would put her beside us in her wheelchair, set up the table and everything for her, and tell her, ‘Don’t eat it yet; wait until they announce the call to prayer.’ Then she thought that she had been fasting. But she could not pray and do those things, so we would give her the Qur’an and say, ‘Look,’ and then she would trace the lines with her finger.”

Caregivers' efforts to support the patient's emotional well-being were shaped by what they knew about the individual patient rather than by general strategies. P10 chose books and songs the patient herself liked and shielded her from visitors she disliked. P9 included the patient in family gatherings, dressing him well to prevent feelings of rejection. P13 arranged weekly outings despite the difficulty of transporting a wheelchair, and found a way to include her mother in Ramadan by serving her meal at iftar time and giving her the Qur'an to touch and read. Caregivers also managed their own emotions in order to continue caring. After becoming frustrated, P5 left the house for ten minutes and then returned to the patient's exercises, and she recommended humour and flexibility to others. Together, these accounts show that caregivers recognised what mattered to the patient, adapted their strategies to his or her preferences and responses, and regulated their own reactions. This is the emotional adaptability and situational judgement that distinguishes competent caregiving from routine companionship.

### 3.4. Preparedness and knowledge updating

This category represents the updating of caregivers’ competencies and capabilities through active and continuous learning to prepare for potential problems. In this regard, caregivers continuously and independently sought to acquire and update the knowledge and skills required to manage critical situations and potential complications of stroke. This competency was manifested in three characteristics: vigilance and follow-up of complications and acute problems, monitoring the patient’s blood pressure under different conditions, and continuity of learning and knowledge updating.

#### 3.4.1. Vigilance and follow-up of complications and acute problems

To respond to potential changes and problems in the patient’s condition, caregivers continuously monitored the patient and, when unexpected symptoms occurred, drew on their knowledge and experience to identify changes and sought guidance from specialists for problem-solving. They followed the problem until the patient’s condition stabilised and, when circumstances changed, modified their caregiving actions accordingly. Previous experiences with problems also helped them become better prepared for similar situations.

Participant 4 stated:

“For example, I would wake up every half an hour and check on my mother. I was constantly checking on her and always paying attention to whether she needed anything. Because she couldn’t speak, I was worried that she might become upset. I tried to stay in the same room where my mother was. I brought her into my room so that even when I wanted to do my work on the computer, I could keep an eye on her and stay beside her.”

For example, Participant 2 emphasised that caregivers should always remain vigilant to prevent accidents. She stated:

“For example, I say that we have to be vigilant as caregivers. We have to pay attention. For example, to her medications... We have to be alert in case she wants to get up. At night, I would even leave the light on. If she wanted to go to the bathroom, I would hold her hand. I would turn on the bathroom light. I would put her slippers there. I would hold her hand. I say that we have to be vigilant so that nothing happens to her.”

Participant 5 emphasised the importance of asking questions and seeking guidance in uncertain situations:

“Whenever something comes up, I ask. Because I don’t know, and I don’t want to do something blindly... Anything I’m unsure about, I ask about.”

Caregivers also assessed the patient’s condition when they observed sudden changes and decided on an appropriate course of action based on the circumstances. Participant 10 stated:

“Suddenly, she feels unwell, she becomes dizzy, or she herself says that she doesn’t feel well, her speech becomes slurred, for example, her hands become cold... We realise that something is not right, so we bring the blood pressure monitor and check her blood pressure.” (Participant 10)

By continuously monitoring the patient’s elimination status and paying attention to abnormal changes in urine characteristics and accompanying symptoms, caregivers also played a role in the timely identification of problems and follow-up of treatment. Participant 14 stated:

“Honestly, when I was emptying her urine bag, I felt that there was something unusual about her urine. It was neither yellowish nor brownish; it was just different. She also had a fever for a day or two... At first, I thought she had a gynaecological infection. Then, when her doctor came, he said, ‘No, she has a urinary tract infection.’ He prescribed strong IV fluids, the kind they give in the hospital, and they came to the house to administer them.”

Vigilance in this subcategory was not simply constant presence; it was purposeful, anticipatory monitoring shaped by the patient's limitations. Because P4's mother could not speak, P4 moved her into her own room so that changes in the patient's needs or mood could be noticed without verbal communication. P2 prepared the environment in advance (leaving the light on, placing slippers, holding the patient's hand on the way to the bathroom) to prevent accidents. Caregivers also recognised the limits of their own knowledge: P5 sought professional guidance in uncertain situations rather than acting “blindly.” When warning signs appeared, they interpreted them and acted. P10 recognised cold hands and slurred speech as abnormal and checked blood pressure. P14 noticed an unusual change in urine together with fever, informed the physician, and followed the treatment, which led to the diagnosis of a urinary tract infection.

Together, these accounts show that caregivers recognised deviations from the patient's usual state, judged their significance, chose an appropriate action, and coordinated with health professionals, which is how competency in managing complex situations was enacted.

#### 3.4.2. Monitoring the patient’s blood pressure under different conditions

Caregivers monitored the patient’s blood pressure and heart rate according to the patient’s condition and under different circumstances, drawing on their knowledge and experience. They first carefully observed and assessed the patient’s condition and, when they noticed physical or behavioural changes or unusual signs, monitored vital signs, including blood pressure. Some caregivers also modelled their practice on that of nurses and monitored vital signs at home before administering certain medications.

One participant stated:

“Over these years, I’ve learned to tell from his face, his pulse, and his eyes whether his blood sugar or blood pressure is high. I’ve learned how to recognise it. When his blood pressure drops, it seems as though his eyes are closing and he becomes lethargic, so I check his blood pressure.” (Participant 9)

Another participant described monitoring the patient before administering medications:

“We have to do this... I definitely have to check his blood pressure and his heart rate, and then give him his medications. Since I stayed in the hospital myself, I saw the nurses checking his heart rate and blood pressure before giving him the medication... For example, if his heart rate was low or his blood pressure was low, he shouldn’t take the medication. We learned these things through experience.” (Participant 3)

Therefore, competency in this category was not limited to the technical skill of measuring blood pressure; rather, it involved the integration of knowledge, practical skills, observation, and situational judgement, reflecting caregiving competency.

#### 3.4.3. Continuous learning and updating

Caregivers experienced learning as a continuous process driven by the changing needs of the patient. To acquire and update their knowledge and practical skills, they sought information from physicians, nurses, rehabilitation specialists, the experiences of other families, and online resources. This learning was shaped in response to real caregiving problems, and caregivers attempted to apply what they had learned in the home environment and modify their practices according to the patient’s condition.

Participant 6 described continuous learning of rehabilitation exercises:

“I mostly try to learn those exercises and movements. In our city, I only knew three exercises: a bicycle exercise, one for the arms, and ordinary walking. But here, since I came to this hospital, I have learned more than 50 movements. I learned them and now I do them with my father. If I have any problem, I ask the doctor or the student who teaches the exercises. They answer my questions. I like learning.”

Another participant described learning techniques to support speech rehabilitation:

“For example, the pronunciation, the shape of the lips, and the positions the lips take when pronouncing words, so that he can repeat them. He looks in the mirror, and she also looks at me. He looks at his own lips in the mirror to see them and see his facial expressions, and tries to do the same. We had learned these things from speech therapy. We practiced these exercises with his for some time.” (Participant 3)

When caregivers did not receive sufficient education in the hospital, they searched for online resources and used specialised online consultation services to obtain the information they needed to care for the patient. Participant 7 stated:

“They didn’t tell us anything about it at the hospital, so we kept searching the internet and reading about what we should do. The internet really helped us a lot. There is a website called AMD, if I’m not mistaken. My husband pays for it; he pays a few dollars a month, and they answer whatever questions we have. There are doctors there who help us.” (Participant 7)

Participant 4 also stated:

“I had searched for some exercises on YouTube... I searched for everything. I also searched in English so that it would be correct and according to proper principles.” (Participant 4)

Preparedness was not simply being present with the patient; it involved monitoring, interpreting changes and learning in response to real problems. Caregivers watched for early warning signs and acted on them. P10 recognised cold hands and slurred speech as abnormal and checked blood pressure, and P14 noticed an unusual change in urine together with fever and informed the physician, which led to the diagnosis of a urinary tract infection. Caregivers also developed their own ability to read the patient's state. P9 learned to recognise changes in blood pressure from the patient's face and eyes, and P3 learned from nurses to check heart rate and blood pressure before giving medication. When information was lacking, they sought it actively. P6 learned more than 50 exercises in the hospital, P3 learned speech-therapy techniques such as using a mirror to show lip positions, and P7 and P4 searched the internet, used paid online consultation (P7) and checked English-language sources for reliability (P4). P5 asked professionals when unsure rather than acting “blindly.”

Together, these accounts show caregivers recognising deviations, judging their significance, choosing an action, coordinating with health professionals and updating their knowledge as needs changed. This reflects competency as continuous learning and the management of complex situations, and learning was part of problem-solving rather than a separate activity.

### 3.5. Support for recovery of cognitive functioning

This category represents caregivers’ competency in providing support for the recovery of patients’ cognitive functioning. Caregivers’ experiences indicate that family caregivers play an active role in patients’ cognitive recovery. In the absence of patients’ communication abilities, they participate in the cognitive recovery process through patience, creativity, and emotional support by understanding patients’ needs and providing an appropriate context for strengthening memory and attention. This competency was characterised by caregivers’ continuous efforts to establish communication and strengthen their patients’ cognitive abilities.

#### 3.5.1. Efforts to establish communication

When faced with aphasia or an inability to speak or express needs, caregivers used alternative methods to maintain and strengthen communication. Rather than relying on a single fixed approach to communication difficulties, caregivers managed communication problems by trying different strategies, interpreting the patient’s responses, and adapting their communication methods to the patient’s abilities. Depending on the patient’s capabilities, they used writing, a whiteboard, gestures, sequential questioning, and repetition, and when one method was ineffective, they adopted another approach.

Participant 13 described using a whiteboard and converting letters into words and sentences:

“Sometimes she wanted to say something, but we couldn’t understand her. We would bring a whiteboard. We had written the letters on it, and then we would turn the letters she meant into words and the words into sentences, to see exactly what our mother wanted... because she couldn’t write.”

Participant 4 also used extensive questioning and interpretation of the patient’s nonverbal responses to establish communication:

“Of course, she couldn’t and still can’t speak... We would somehow proceed with twenty questions... She could nod her head and gesture... I would stay beside her for an hour... to understand what she was saying.”

#### 3.5.2. Strengthening cognitive abilities

Caregivers used various strategies to stimulate and maintain patients’ memory, attention, and recall abilities, including continuous conversation, questioning, repetition of information, flashcards, a whiteboard and marker, family photographs and videos, cognitive games, and everyday activities. Exercises were gradually adjusted according to the patient’s abilities and responses, and caregivers attempted to integrate cognitive activities into everyday life. Participant 7 described the use of repetition and continuous conversation to stimulate the patient’s attention and recall:

“When I would say to her, ‘Maryam, Maryam, Maryam,’ she couldn’t even understand her own name. But I said ‘Maryam’ and repeated it so many times that she eventually recognised her name... I would talk to her continuously. ‘Maryam, look here. Maryam, raise your hand...’ I was constantly talking to her.”

The same participant gradually progressed the exercises to strengthen the patient’s cognitive abilities, beginning with simple activities and extending them to everyday situations:

“At home, we made flashcards of the letters for her, along with number flashcards... We worked with her from 1 to 10... After she learned that, we moved on to the license plates of cars... We would ask her, ‘What number is on this car’s license plate? Tell me the first letter.’... Then, when she answered, we encouraged her, until we taught her the numbers and the letters.”

These experiences demonstrate the dynamic and problem-oriented nature of cognitive caregiving, whereby caregivers selected appropriate strategies according to the patient’s specific difficulties and modified the level and method of practice based on the patient’s responses.

Participant 4 also described using a whiteboard to retrain the patient’s knowledge and writing ability:

“During the first week when I was in the hospital, I got a whiteboard and a marker. Then I would tell her to write. At first, she couldn’t remember anything at all... I would teach her. For example, I would say, ‘Your husband’s name is Ali.’ I would teach her ‘Ali,’ and the second time, she would write it herself. And the interesting thing was that, gradually, these words started coming back into her mind. I noticed that she could eventually read texts.” (Participant 4)

Caregivers also attempted to maintain patients’ active participation by involving them in everyday activities and creating opportunities for them to use their remaining abilities, thereby helping to prevent further decline in their functional capacities. Participant 13 described how she deliberately involved her mother in cooking activities:

“I would put her next to me in the kitchen in her wheelchair, and deliberately say, ‘Mom, I want to make this food, but I don’t know what to mix with what.’ Then she would show me by gesturing. Even though she knew that I might actually know better than she did, she would still show me. We didn’t miss these moments, not even for a moment.” (Participant 13)

Supporting cognitive recovery, as caregivers lived it, was less a set of exercises than a continuing effort to reach the person behind the impairment and to keep them involved in daily life. Where speech was lost, caregivers did not wait for a therapist's instruction; they built ways of understanding the patient themselves, turning letters into words and words into sentences on a whiteboard (P13), or working through “twenty questions” and reading nods and gestures for an hour at a time (P4). When one approach failed, they tried another, so communication became something they worked out through trial and attention to the patient's responses. The same adaptive logic shaped cognitive stimulation. P7 repeated a name until the patient recognised it, then moved step by step from letter and number cards to car license plates, and P4 rebuilt writing by teaching one familiar word at a time, each stage set by what the patient had just managed. Caregivers also protected the patient's role and dignity: P13 asked her mother to show her how to cook, leaving the patient with something real to contribute. Together, these accounts show competency as the ability to interpret a patient who cannot speak, to adjust the level of challenge to the patient's progress, and to build recovery into ordinary family life.

## 4. Discussion

The findings of this study showed that caregiving competence among family caregivers of older adults with stroke is a multidimensional, dynamic, and context-dependent phenomenon that extends beyond the performance of routine caregiving tasks. The five identified dimensions included Management of Safety and Motor Functioning; Control of Essential Intake and Elimination Processes; Maintenance and Promotion of Psychological and Emotional Well-Being; Preparedness and Knowledge Updating; and Support for the Recovery of Cognitive Functioning. This finding is consistent with previous studies regarding the multidimensional nature of caregiver competence (Cheng & Chau, 2017). Furthermore, reviewing other studies on caregiving competence confirms its multidimensional nature.(Jung et al., 2022; Purba & Sutharangsee, 2014; Sanches et al., 2021; Wang et al., 2025).

Caregivers integrated these dimensions simultaneously into their daily activities; they responded to the patient’s physical, psychological, and emotional needs, acquired new skills, and developed preparedness to deal with potential problems. Therefore, competence was manifested not through mastery of a single skill, but through the combination and integration of knowledge and practice in real-life caregiving situations. This finding is consistent with studies reporting family caregivers’ involvement in the physical care, rehabilitation, psychological support, and management of daily activities of patients with stroke (Hesamzadeh et al., 2017; Pourhasan & Vasli, 2025).

In the present study, competence had a dynamic and evolving nature. Caregivers often entered the caregiving role with limited knowledge and preparedness and gradually developed greater practical knowledge, situational judgement, and adaptive capacity through repeated interaction with the patient, direct experience, observation, trial and error, problem-solving, and modification of previous strategies. They adjusted, for example, food consistency, exercise intensity, or environmental conditions according to changes in the patient’s condition. This finding is consistent with the concept of “learning by doing,” whereby caregivers of patients with stroke gradually acquire caregiving knowledge and strategies through everyday experience and do not rely solely on formal education (Cameron & Gignac, 2008; Greenwood & Mackenzie, 2010). The present study confirms this finding but extends it by demonstrating that learning through practice was not a one-time process, but rather an active (self-directed) and continuous process. Caregivers repeatedly evaluated the patient’s responses, tried different approaches, and modified their actions according to changing needs; they learned not only what to do, but also when and how to adapt their actions to the patient’s changing circumstances.

This process can also be explained within the framework of Benner’s theory of competency development. At the beginning, caregivers faced limited experience and uncertainty, but through repeated exposure to caregiving situations, observation, practice, trial and error, and attention to the patient’s responses, they moved toward more complex practical knowledge and situational judgement (Benner, 1984). Therefore, in this study, competence was not a fixed characteristic, but rather a developmental process from initial, rule-dependent performance toward more experienced and situation-responsive performance.

The context-dependent nature of caregiving competence was also evident in this study. Caregivers adjusted their actions according to the patient’s functional status and fluctuations in symptoms, family resources, and the characteristics of the home environment. Although domains such as physical assistance, rehabilitation, emotional support, and management of daily activities have also been reported in previous studies (Lobo et al., 2023; Young et al., 2014), and caregivers’ involvement in mobility, daily activities, medication management, nutrition, and rehabilitation is also consistent with previous evidence (Einstad et al., 2022; Grimby et al., 1998; Predebon et al., 2021), the role of caregivers in supporting patients’ emotional adjustment and maintaining interpersonal relationships is also consistent with the findings of Harrison et al. (2017). The present finding shows that these activities were not performed as fixed and standardised behaviours; rather, caregivers continuously interpreted the patient’s needs and adapted their actions to the specific clinical, family, and environmental conditions. Therefore, the present study does not claim that these caregiving domains are entirely new; rather, its main contribution lies in demonstrating how these domains are integrated with continuous learning, situational judgement, and adaptation to changing circumstances in everyday home caregiving. From this perspective, the present findings do not conflict with the previous literature but provide a more process-oriented understanding of how recognised caregiving responsibilities are transformed into competence within the real context of home care.

Participants performed some of their caregiving actions within the framework of family roles, interpersonal commitments, and meaningful cultural and religious activities. For example, some caregivers had developed specific practical strategies to help patients maintain participation in religious and spiritual activities, including adapting assistance for performing prayers and other religious practices despite physical limitations. These actions were not merely extensions of standard rehabilitation instructions; rather, they reflected culturally tailored solutions that caregivers developed to preserve the patient’s valued activities and roles in everyday life. This interpretation is consistent with qualitative research conducted in Iran on family caregivers of stroke survivors, which has shown that caregiving is shaped by cultural commitments and that caregivers use religiosity and social support as resources for adaptation (Hesamzadeh et al., 2017). Therefore, context dependence in the present study included not only clinical and environmental adaptation but also the integration of meaningful cultural practices into everyday caregiving.

One distinctive finding of the present study was the identification of support for the recovery of cognitive functioning as an independent dimension of competence. Although previous studies have reported caregivers’ involvement in physical rehabilitation and daily activities (Neethu & Vayalil, 2025; Viscogliosi et al., 2019), in the present study, caregivers used strategies such as flashcards, board games, memory exercises, structured repetition, and alternative communication strategies. Given the prevalence of cognitive and communication impairments following stroke and the need for repeated stimulation and communication support at home, this finding can be explained accordingly (Shafer et al., 2019). Therefore, cognitive and communication support can be considered a specific component of caregiving competence among caregivers of patients with stroke, which becomes particularly important after the completion of formal rehabilitation sessions (Kokorelias et al., 2020; Liu et al., 2026).

Caregivers’ preparedness and knowledge updating also developed partly in response to the gap between discharge education and the actual needs of home care. Limited post-discharge services and inadequate access to professional guidance led caregivers to independently seek information and draw on experience, family members, peers, and informal sources (Liao et al., 2024). Iranian studies have also reported inadequate preparation before discharge, educational needs after returning home, and concerns related to the transition from hospital to home (Keykha et al., 2022; Merati-Fashi et al., 2022; Noorian et al., 2025). Furthermore, routine hospital education alone was insufficient to reduce caregiving burden, whereas structured supportive home-care programmes accompanied by education and post-discharge follow-up were more effective (Ashghali Farahani et al., 2021). Therefore, self-directed learning in this study can be considered, to some extent, an adaptive response to insufficient support during the transition of the patient from hospital to home. Continuous monitoring of the patient’s condition also demonstrated the active nature of competence. Through observing functional changes, monitoring symptoms, recording vital signs, and identifying warning signs, caregivers gradually acquired skills in practical monitoring and decision-making for managing problems at home. This did not imply assuming the role of a health professional, but rather reflected the gradual development of practical skills through experience, information seeking, and interaction with healthcare providers and peers; a finding that is consistent with reports of unmet educational needs and caregivers’ ongoing efforts to learn and seek help (Maggio et al., 2024).

Conceptually, the present findings can also be explained by distinguishing between perceived competence and competence enacted in practice. Cheng and Chau (2017), in examining the Chinese version of the Caregiving Competence Scale among 118 family caregivers of stroke survivors, conceptualised competence primarily in terms of perceived ability, feelings of competence, and caregivers’ confidence (Cheng & Chau, 2017). In contrast, the present study focuses on the content-based and observable characteristics of competence, that is, the domains that caregivers actually employed in practice to respond to patients’ needs. This distinction suggests that competence can be considered not merely as a sense of ability, but as a practical capacity to integrate knowledge and skills, interpret situations, adapt strategies, and act appropriately according to patients’ needs.

The specific characteristics of the stroke disease trajectory also help explain this pattern. The sudden onset of the disease may place families in the caregiving role with limited preparedness (Abdul Rashid et al., 2026). The rehabilitation-oriented nature of the disease extends caregiving beyond the provision of basic needs and requires continuous participation in functional recovery (Grabowska-Fudala et al., 2026). Furthermore, the co-occurrence of motor, cognitive, communication, and emotional impairments requires caregivers to integrate multiple domains of competence simultaneously (Rajda et al., 2025). Alongside family, cultural, and structural conditions of home care, these characteristics explain why caregiving competence develops as an adaptive and evolving capacity.

Overall, the three characteristics of multidimensionality, dynamism, and context dependence should be considered interrelated features of caregiving competence. Multiple dimensions define the scope of caregiving needs; experience and continuous learning contribute to the development and transformation of competence over time; and the patient’s condition, family resources, and home environment determine how these competencies are manifested in practice. Accordingly, caregiving competence among older adults with stroke can be conceptualised as an integrated capacity to identify needs, learn from experience, adapt strategies, and act appropriately under specific home-care conditions.

## 5. Limitations

In the present study, efforts were made to minimise researcher bias. To this end, an inductive approach was used for conceptualisation, semi-structured interviews were conducted, and data analysis was grounded in the experiences and accounts of the participants. In addition, member checking and external checking were employed to enhance the credibility of the findings, and an audit trail was maintained throughout all stages of the study.The findings of this study reflect the experiences of Iranian family caregivers of older adults with stroke, including both ischaemic and haemorrhagic stroke. These findings may be transferable to family caregivers of individuals with stroke in other settings, depending on similarities in cultural and social contexts and caregiving conditions. However, their applicability to caregivers of individuals with other chronic conditions should be considered cautiously and assessed in light of the specific characteristics of each caregiving context.

## 6. Conclusion

Caregiving competency among family caregivers of older adults with stroke is a multidimensional, dynamic, and context-dependent phenomenon that develops gradually through interaction with the patient, experience, trial and error, problem-solving, and continuous learning. Caregivers often enter the caregiving role with limited preparedness and subsequently develop their competencies across different dimensions through everyday experience, experiential learning, and situational judgement. Their competency is continuously shaped by patients’ specific needs and problems, including the need to support cognitive recovery, changes in the patient’s condition, the availability of family and professional support, and the limitations and resources present in the home environment. The findings of this study indicate that the identified dimensions of caregiving competency are not independent characteristics or mechanically separate components of a system. Rather, they are integrated, continuous, and organically interconnected within an open system. For example, Preparedness and Knowledge Updating serves as a source of knowledge that supports the development and enactment of competency across the other dimensions, including Management of Safety and Motor Functioning, Control of Essential Intake and Elimination Processes, Maintenance and Promotion of Psychological and Emotional Well-Being, and Support for Recovery of Cognitive Functioning. Similarly, vigilant monitoring and assessment, situational judgement, and the search for appropriate solutions, which reflect problem-solving skills, are embedded within and operate across all five dimensions of caregiving competency.

## 7. Strengths and limitations of the study

These findings may have important implications for the health system. Given that caregivers often enter the caregiving role with limited preparedness, education and preparation for assuming caregiving responsibilities across the five identified dimensions could begin before hospital discharge and continue after discharge. Accordingly, the identified dimensions may provide a basis for developing context-specific frameworks of caregiving competency for family caregivers of older adults with stroke and for designing educational and supportive programs tailored to their needs. Furthermore, these findings may serve as a useful conceptual guide for developing a specific instrument to assess caregiving competency among family caregivers of older adults with stroke—an instrument that specifically measures caregiving competency itself rather than merely caregivers’ perceived confidence, self-efficacy, or caregiving outcomes.

## Supplementary Material

Supplementary File 1.doc Supplementary File 1.doc

## Data Availability

The datasets generated during and/or analysed during the current study cannot be shared due to ethical, privacy or security concerns.
