# Supplementary material for: Experiences of family caregivers of older adults with stroke regarding caregiving competency: a qualitative study
Source: Int J Qual Stud Health Well-being. 2026 Nov 4;21(1):2759356. doi: 10.1080/17482631.2026.2759356 (PMC13757909; doi:10.1080/17482631.2026.2759356)
Supplement: Supplementary file 1 [file ZQHW_A_2759356_SM3787.doc]

**Interview Guide Questions**

At the beginning of the interview, the interviewer welcomed the participant and thanked them for their willingness to participate in the study. The interviewer briefly explained the purpose of the study, the confidentiality of the information provided, and the voluntary nature of participation. To establish initial rapport and create a comfortable and relaxed environment for discussion, a general opening question was then asked.

**Warming Question**

“Thank you for agreeing to talk with me in this interview. Before we begin, could you please tell me a little about yourself and your family?”

**Interview Questions**

Please describe what you knew about caring for the patient from the time they were transferred from the hospital to the home.

What did you learn about caring for your patient, and from whom did you learn it?

What did you do to care for your patient at home?

If you encountered difficulties while caring for your patient at home, what did you do?

In addition to physical care, did you provide psychological and emotional care? If so, how?

**Probing Questions**

“Could you tell me a little more about that?”

“Could you give me an example?”

“Could you explain what you mean by that in more detail?”

“What did you do in that situation?”

**Final Question**

“Thank you for participating in this interview. Is there anything else you would like to share about your experiences of caring for a person with stroke?”
